# Supplementary material for: RASSF1A controls tissue stiffness and cancer stem‐like cells in lung adenocarcinoma
Source: EMBO J. 2019 May 27;38(13):e100532. doi: 10.15252/embj.2018100532 (PMC6600643; doi:10.15252/embj.2018100532)
Supplement: Supplementary file 1 — Expanded View Figures PDF [file EMBJ-38-e100532-s001.pdf]

Expanded View Figures

Figure EV1. RASSF1A suppresses formation of primary tumours and metastatic progression in lung adenocarcinoma.

A Representative longitudinal MR images of a lung tumours in an individual mouse at day 30 formed by $H1299^{control}$ or $H1299^{RASSF1A}$ overexpressed cells. Red arrowheads indicate primary tumours in ipsilateral (left) lungs. Bottom panel: Representative images of macroscopic appearances of tumour nodules on the lung surface at day 30, identified as patchy and whitish areas.

B Representative images of the lung primary tumours generated at day 17 after orthotopic lung injection with either $H1299^{control}$ or $H1299^{RASSF1A}$ cells, and arrows indicate lung primary tumours in ipsilateral (left) lungs (n = 4 mice per group).

C Representative fluorescence images of $H1299^{control}$ and $H1299^{RASSF1A}$ primary lung tumours stained for macrophages (green) with F4/80 and DAPI (blue). Images are presented as merge F4/80 with DAPI. Scale bars: 100 µm.

D Western blot analyses of pYAP1 protein expression in HOP92 cell lines after stable knockdown of RASSF1A by shRNA lentiviral transfection. Right: Resazurin assay used for analysing proliferation ratio in $HOP92^{shcontrol}$ and RASSF1A knockdown $HOP92^{shRASSF1A}$ cells shows great increased in proliferation when expression of RASSF1A protein was impaired. Statistical analyses were performed using Student's *t*-test 2-(tailed) of n = 2 experiments, and error bars represent the mean ± SEM.

E Left: Graph showing size of primary tumours formed by HOP92 lung adenocarcinoma cells after lung orthotopic injection, day 30. Knockdown of RASSF1A in HOP92 cells (shRASSF1A) (n = 7 mice per group) demonstrated higher ability to bear primary tumours that control group, when RASSF1A was expressed (shcontrol) (n = 6 mice per group). Right: Representative longitudinal MR images of a lung tumours in an individual mouse at day 30 generated by $HOP92^{shcontrol}$ (n = 6 mice per group) or $HOP92^{shRASSF1A}$ (n = 7 mice per group) by orthotopic lung injection.

Source data are available online for this figure.

© 2019 The Authors

© 2019 The Authors

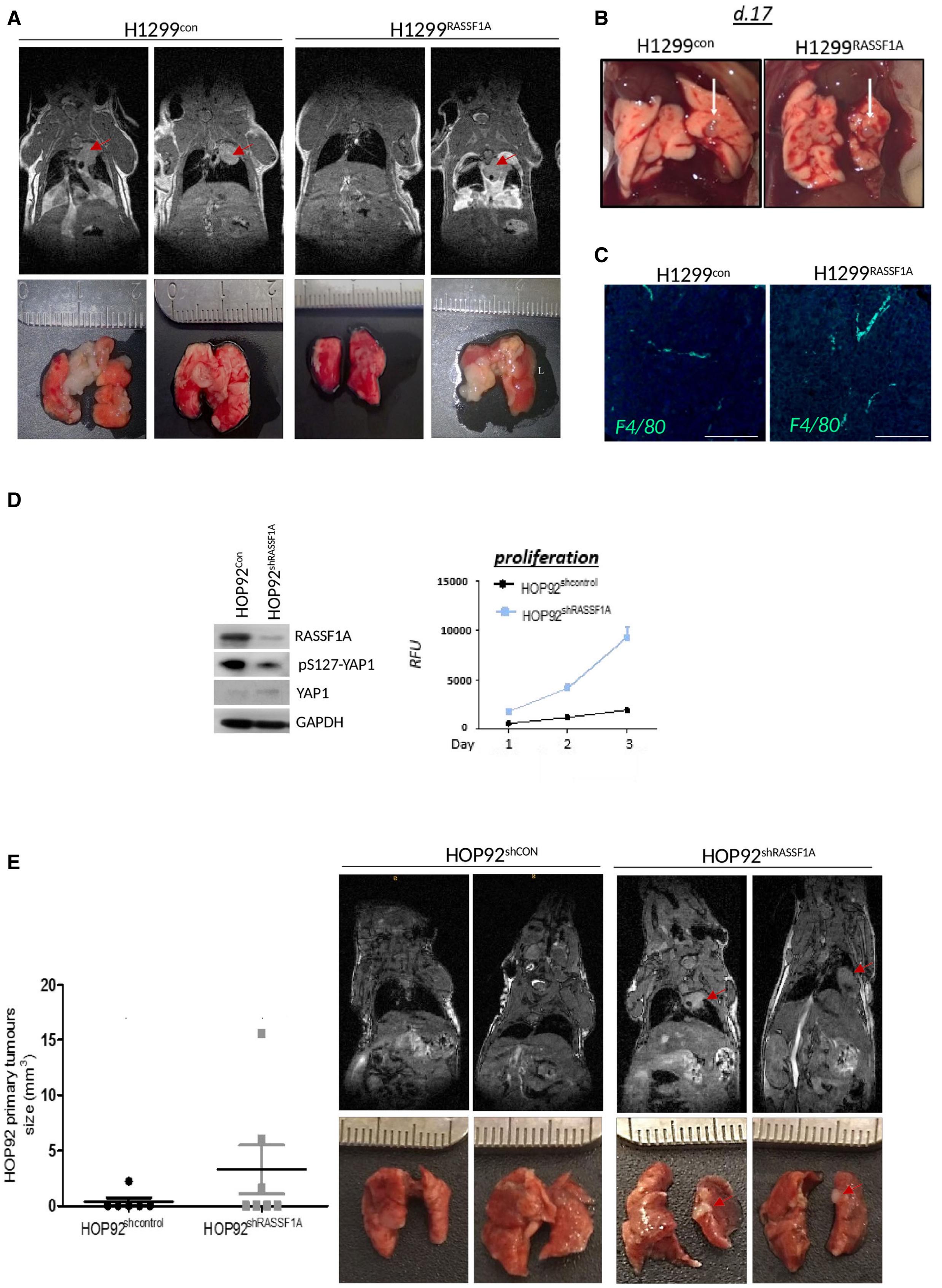

Figure EV1.

 © 2019 The Authors

Figure EV2. Expression of P4HA2 is associated with poor prognosis in solid cancers.

A Kaplan–Meier plots showing overall survival prognosis in breast cancer patients with high and low mRNA expression of TFPI2 and TIMP1 proteins revealed by proteomics study from extracted ECM produced by H1299^{RASSF1A} lung cancer cells. Data show that breast cancer patients with high mRNA expression of TFPI2 and TIMP1 have better prognosis. The *P* values were derived from a log-rank test.

B Clinical outcome and percentage of survival in patients across various cancers show effect of low versus high expression levels of mRNA P4HA2. Data collected from TCGA. The *P* values were derived from a log-rank test.

C Quantification of fluorescence intensity of P4HA2 expression in H1299 cells with or without P4HA2 knockdown, 1.4DPCA treatment or combination of both. Bottom graph: Representative immunofluorescence images showing different expression of P4HA2 and collagen I in H1299^{control} or H1299^{RASSF1A} re-expressing cells. Treatment of H1299^{control} cells with siRNAP4HA2, P4HA inhibitor 1.4-DPCA (inh.) or combination of both shows decreased collagen I expression. Scale bars: 10 μm.

D RT–PCR analysis of relative mRNA expression levels of P4HA2 in H1299 cells validating its after siP4HA2 knockdown.

© 2019 The Authors

A

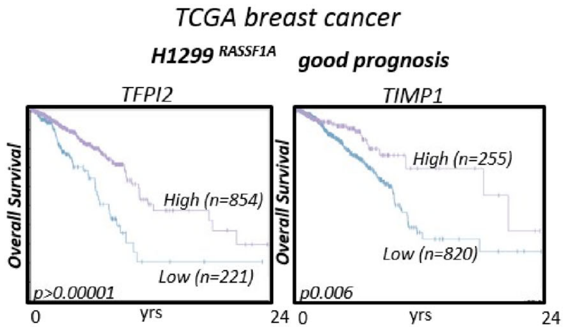

B

TCGA

P4HA2	Expression cutoff FPKM	5yr survival High	5yr survival Low	Log Rank pvalue
Lung (LUAD)	9.2	(n=225) 27%	(n=275) 49%	0.008
Lung (LUSC)	11.7	(n=125) 34%	(n=369) 52%	0.005
CRC	9.7	(n=114) 34%	(n=324) 71%	0.000187
Kidney (KIRP)	7.1	(n=66) 52%	(n=219) 82%	0.000002
Urothelial	6.9	(n=147) 30%	(n=48) 48%	0.000312
Cervical	6.5	(n=171) 54%	(n=120) 81%	0.0000124
HNSCC	6.5	(n=388) 42%	(n=111) 58%	0.00162
Liver	4.8	(n=123) 31%	(n=242) 54%	0.00116
Kidney (KICH)	4.6	(n=13) 61%	(n=51) 91%	0.014
Glioma	3.7	*(n=32) 0%	*(n=121) 10%	0.00269
Stomach	3.2	(n=279) 28%	(n=75) 66%	0.025

C

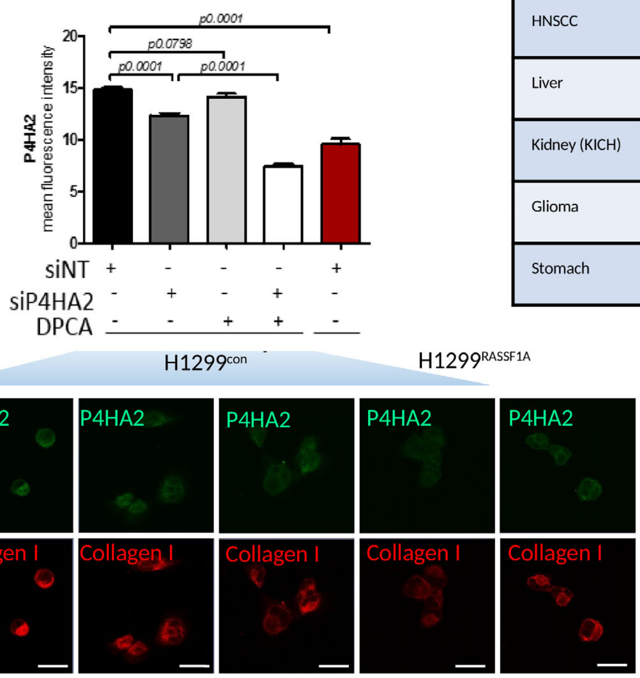

D

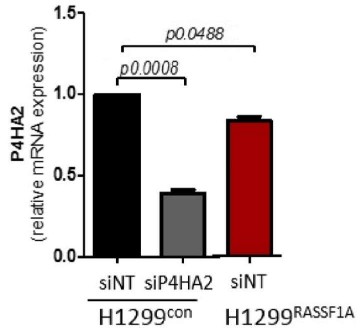

Figure EV2.

© 2019 The Authors

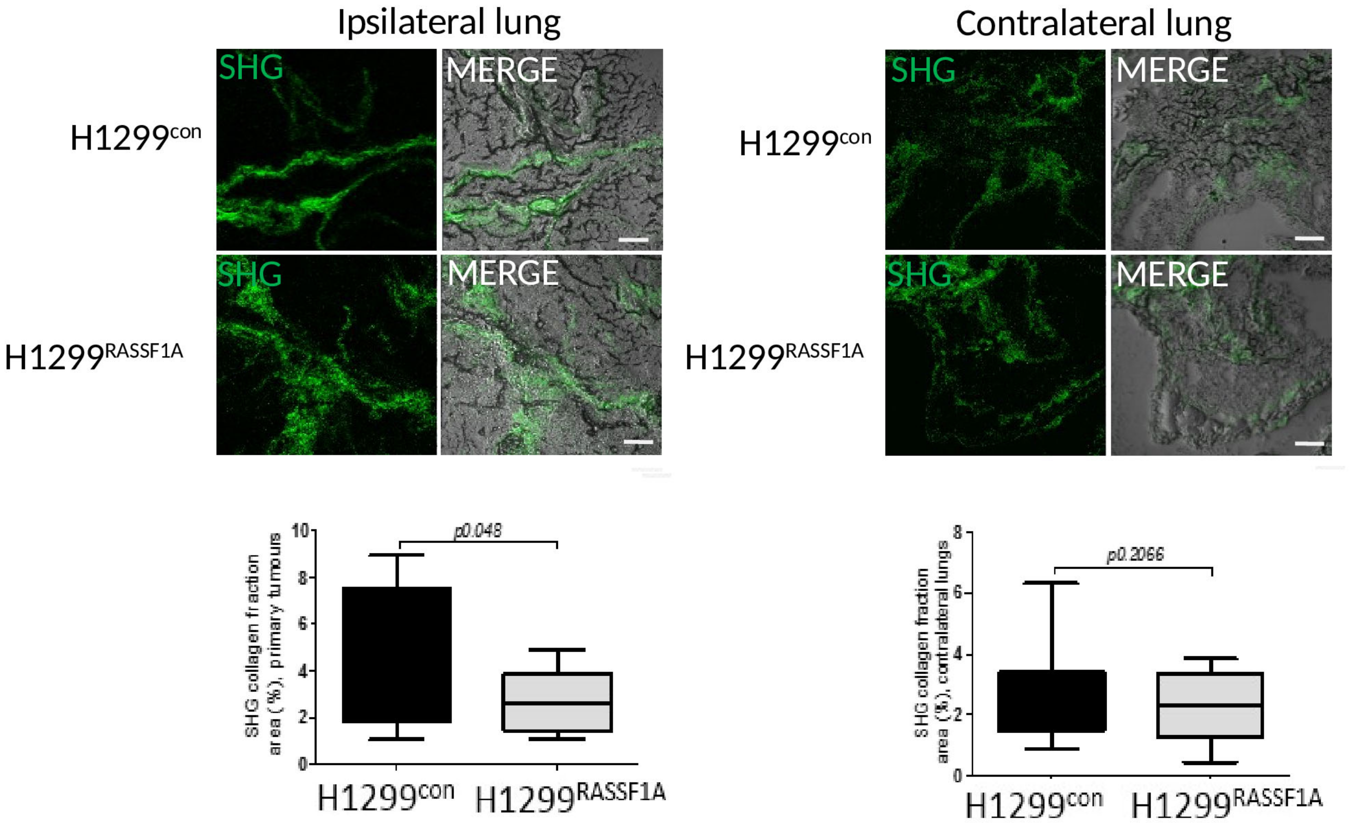

Figure EV3. Mechanical properties of extracellular matrix.
Representative second harmonic generation (SHG) images with quantification of collagen fractions (bottom graphs) in ipsilateral primary tumours (left lungs) and contralateral (right) lungs at day 17, formed by $H1299^{control}$ or $H1299^{RASSF1A}$ re-expressing cells. Scale bars: 50 μm. Statistical analyses were performed using 2-tailed Student's *t*-test. Error bars represent SEM.

Figure EV4. Mechanical properties of extracellular matrix are important for Nanog expression and its nuclear translocation.

A Left: Quantification of Nanog nuclear localization (Pearson's coefficient) in $H1299^{control}$ or $H1299^{RASSF1A}$ (right) overexpressed cells grown in 3D matrix with different collagen concentration. Graphs show that increased collagen concentration of ECM overrules activated Hippo pathway by RASSF1A expression and stimulate Nanog translocation into nucleus. Effect of ECM stiffness on Nanog localization in H1299 cells is compared with cells grown in the plastic (referred as 2D, last bars in graphs). *P* values were determined by 2-tailed Student's *t*-test of $n = 3$ experiments, and error bars represent the mean ± SEM. Bottom: Representative immunofluorescence images of Nanog expression and its localization in $H1299^{control}$ or $H1299^{RASSF1A}$ cells growing in three-dimensional matrix with different collagen concentration. Merge images are combination of Nanog (green) with DAPI (blue). Scale bars: 10 μm.

B Graph bars of quantification of Nanog distribution within H1299 three-dimensional spheroids. Immunofluorescence Nanog signal was apparent either in central part of $H1299^{control}$ spheroids (referred as core) or was uniformly distributed (referred as uniform) over whole spheroids embedded in collagen matrix (2 mg/ml).

C Representative images of $H1299^{control}$ or $H1299^{RASSF1A}$ three-dimensional spheroids grown in 3D collagen matrix (2 mg/ml) and stained for Nanog and hypoxia marker HIF-1α. Scale bars: 50 μm.

D Western blot analyses of Β-catenin, YAP1 and Nanog expression from H1299 cells grown and isolated from three-dimensional collagen matrix after treatment with P4HA inhibitor 1.4-DPCA.

Source data are available online for this figure.

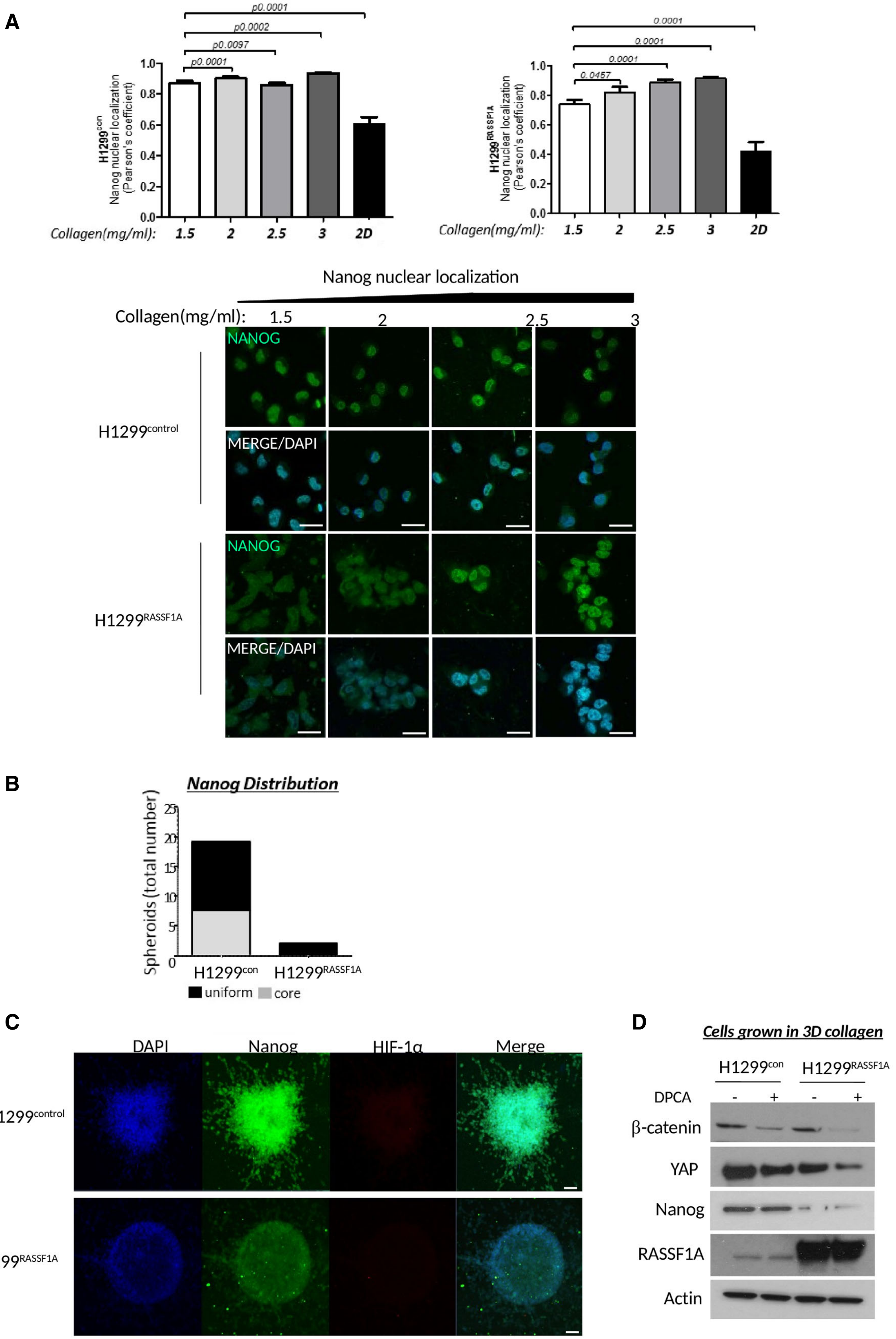

Figure EV4.

 © 2019 The Authors
