# Supplementary material for: RASSF1A controls tissue stiffness and cancer stem‐like cells in lung adenocarcinoma
Source: EMBO J. 2019 May 27;38(13):e100532. doi: 10.15252/embj.2018100532 (PMC6600643; doi:10.15252/embj.2018100532)
Supplement: Supplementary file 2 — Table EV1 [file EMBJ-38-e100532-s002.docx]

| **H1299^control^** | *size of primary tumours (left lung) detected by MRI (mm^3^)* | *No. of surface mets. nodules on left lung* | *No. of surface mets. nodules on right lung* | *weight of left lung*  *(g)* | *weight of right lung*  *(g)* |
| --- | --- | --- | --- | --- | --- |
| *M1* | 10.49 | 4 | 4 | 0.133 | 0.136 |
| *M2* | 0 | 2 | 5 | 0.066 | 0.158 |
| *M3* | 22.23 | 4 | 2 | 0.148 | 0.099 |
| *M4* | 12.9 | 1 | 2 | 0.139 | 0.142 |
| *M5* | 14.04 | 1 | 6 | 0.091 | 0.139 |
| *M6* | 13.05 | 9 | 5 | 0.086 | 0.11 |
| *M7* | 21.98 | 1 | 2 | 0.096 | 0.145 |
| *M8* | 67.199 | 1 | 0 | 0.153 | 0.113 |
| *M9* | 15.77 | 2 | 2 | 0.098 | 0.090 |
| *M10* | 30.24 | 1 | 2 | 0.119 | 0.146 |
| *M11* | 5.73 | 0 | 0 | 0.046 | 0.094 |
| *M12* | 57.26 | 2 | 3 | 0.141 | 0.140 |
| *M13* | 30.8 | 4 | 1 | 0.19 | 0.136 |
| *M14* | 62.38 | 2 | 1 | 0.132 | 0.155 |
| **H1299^RASSF1A^** | *size of primary tumours (left lungs) detected by MRI (mm^3^)* | *No. of surface mets. nodules on left lung* | *No. of surface mets. nodules on right lung* | *weight of left lung*  *(g)* | *weight of right lung*  *(g)* |
| *M1* | 0 | 0 | 0 | 0.057 | 0.098 |
| *M2* | 31.2 | 1 | 1 | 0.086 | 0.069 |
| *M3* | 11.52 | 1 | 1 | 0.065 | 0.127 |
| *M4* | 0 | 0 | 0 | 0.071 | 0.123 |
| *M5* | 18.81 | 1 | 2 | 0.104 | 0.176 |
| *M6* | 0 | 0 | 0 | 0.065 | 0.113 |
| *M7* | 17.73 | 2 | 0 | 0.117 | 0.088 |
| *M8* | 10.09 | 1 | 2 | 0.169 | 0.137 |
| *M9* | 0.00 | 0 | 0 | 0.072 | 0.109 |
| *M10* | 38.45 | 1 | 2 | 0.15 | 0.162 |
| *M11* | 21.60 | 1 | 0 | 0.13 | 0.106 |
| *M12* | 20.79 | 1 | 1 | 0.112 | 0.13 |

Table EV1
