# Supplementary material for: RASSF1A controls tissue stiffness and cancer stem‐like cells in lung adenocarcinoma
Source: EMBO J. 2019 May 27;38(13):e100532. doi: 10.15252/embj.2018100532 (PMC6600643; doi:10.15252/embj.2018100532)
Supplement: Supplementary file 4 — Movie EV1 [file EMBJ-38-e100532-s004.zip › Movie_EV1.docx]

**Movie EV1, Movie EV2.** Representative MRI movies showing lung primary tumours and lung cancer progression on day 30, formed by orthotopic injection of H1299 control or H1299^RASSF1A^ expressing cancer cells. Red-blood&heart, dark blue-lungs, yellow-primary tumours, green,-metastases, cyan blue-oedema.
