# Supplementary material for: RASSF1A controls tissue stiffness and cancer stem‐like cells in lung adenocarcinoma
Source: EMBO J. 2019 May 27;38(13):e100532. doi: 10.15252/embj.2018100532 (PMC6600643; doi:10.15252/embj.2018100532)
Supplement: Supplementary file 6 — Movie EV3 [file EMBJ-38-e100532-s006.zip › Movie_EV3.docx]

**Movie EV3.** Time lapse. H1299^RASSF1A^ 3D spheroids grown and differentiate on Matrigel matrix, 24 hours. Scale bars *200µm*
