# Supplementary figures and images for: RASSF1A controls tissue stiffness and cancer stem‐like cells in lung adenocarcinoma
Source: EMBO J. 2019 May 27;38(13):e100532. doi: 10.15252/embj.2018100532 (PMC6600643; doi:10.15252/embj.2018100532)

**Figure EV1D**

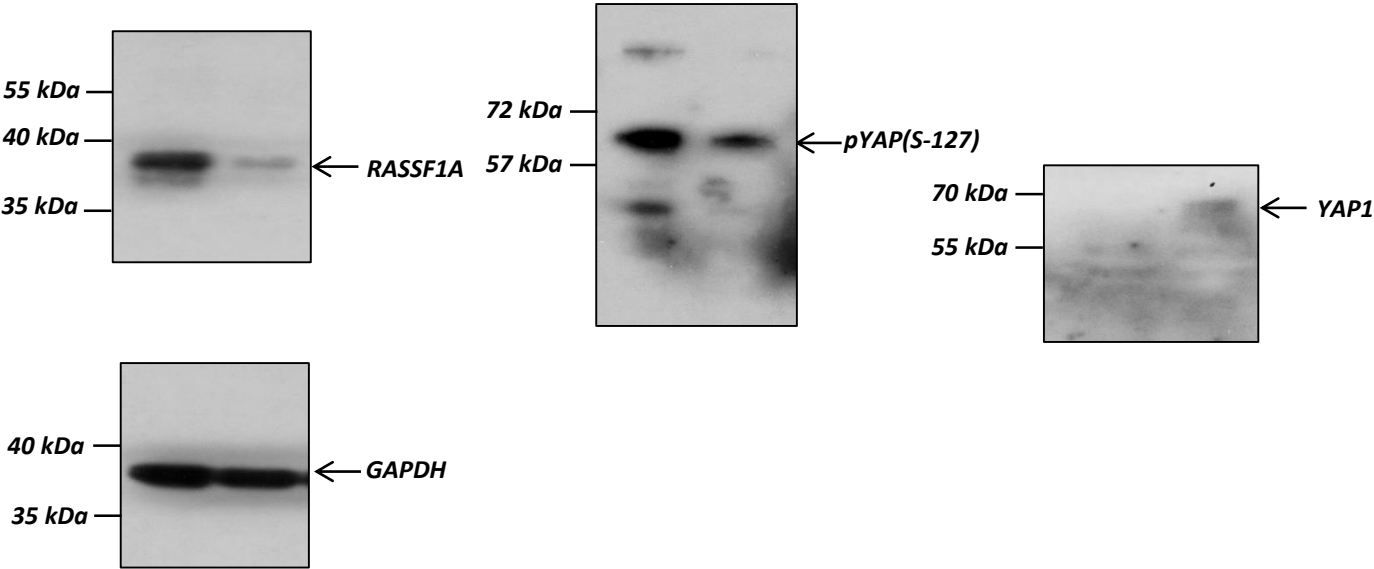

Supplement: Supplementary file 7 — Source Data for Expanded View [file EMBJ-38-e100532-s011.zip › SD_Figure_EV1._uncropped_membranes.pdf]

**Figure EV4D**

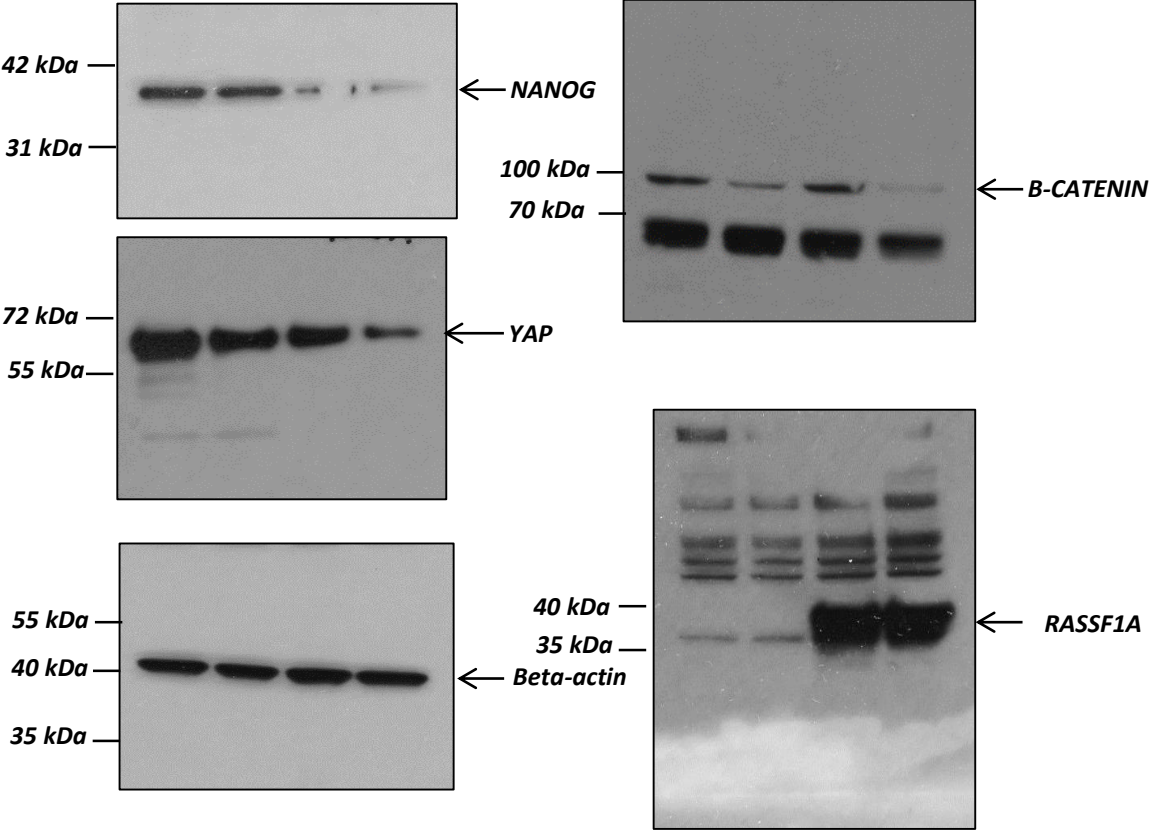

Supplement: Supplementary file 7 — Source Data for Expanded View [file EMBJ-38-e100532-s011.zip › SD_Figure_EV4._uncropped_membranes.pdf]

**Figure 1B**

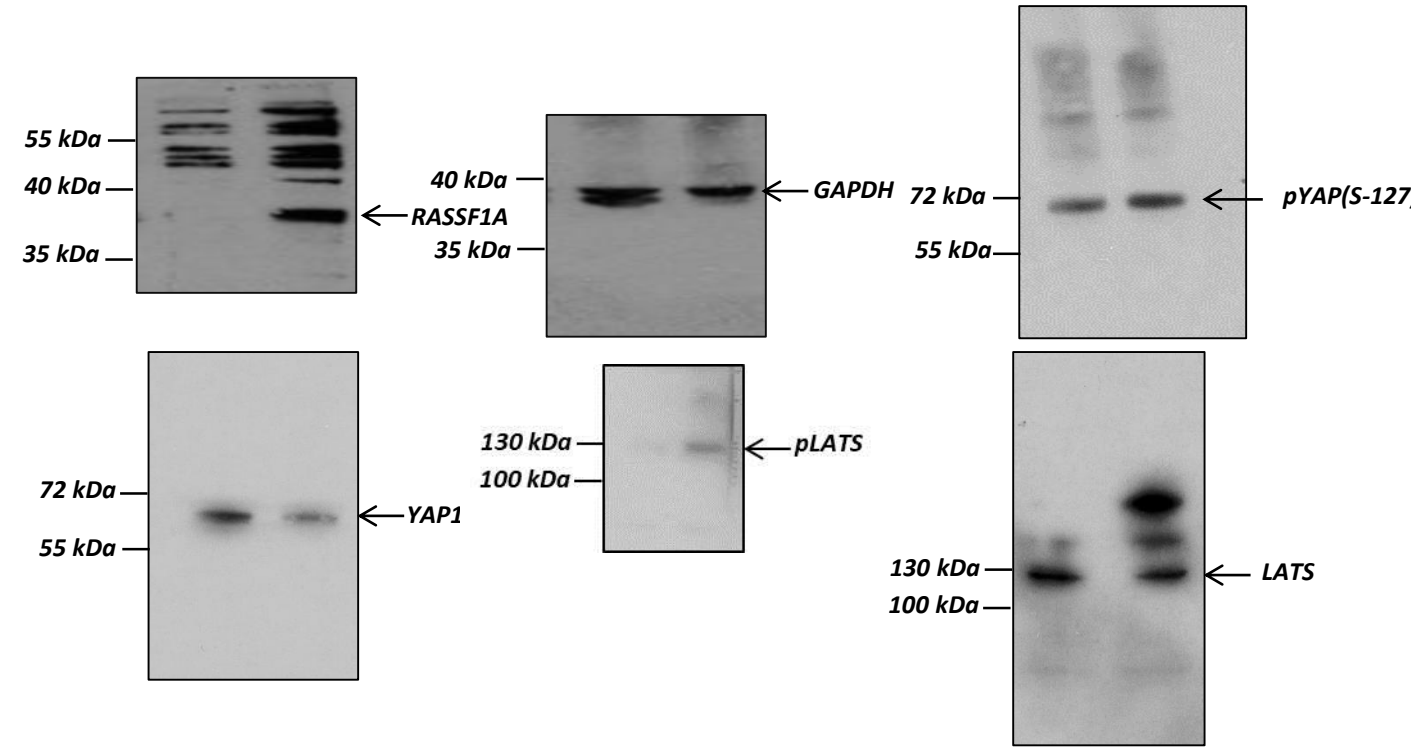

Supplement: Supplementary file 9 — Source Data for Figure 1 [file EMBJ-38-e100532-s007.pdf]

**Figure 2E**

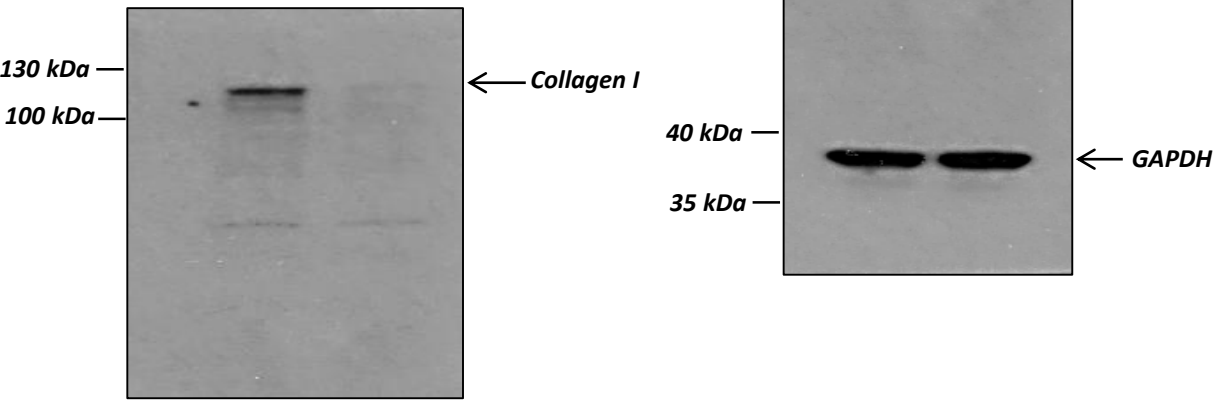

**Figure 2F**

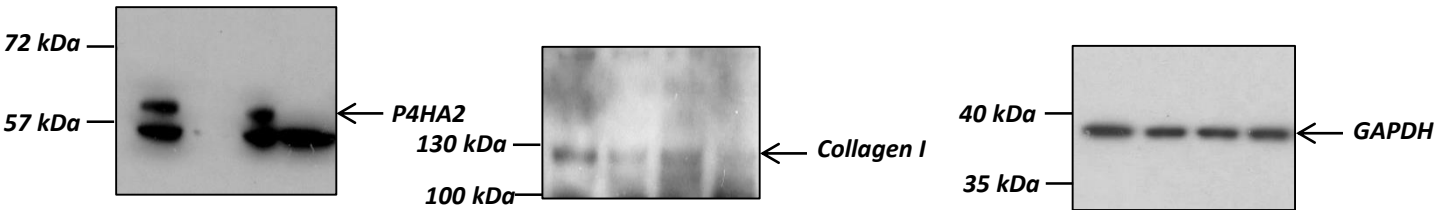

**Figure 2I**

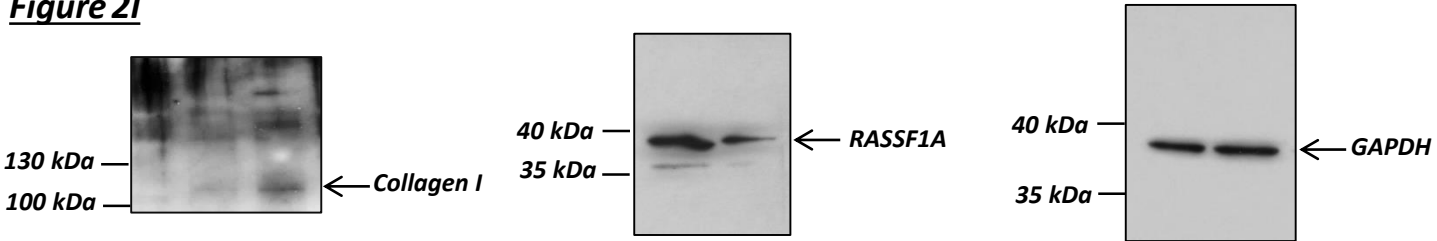

Supplement: Supplementary file 10 — Source Data for Figure 2 [file EMBJ-38-e100532-s008.pdf]

**Figure 6C**

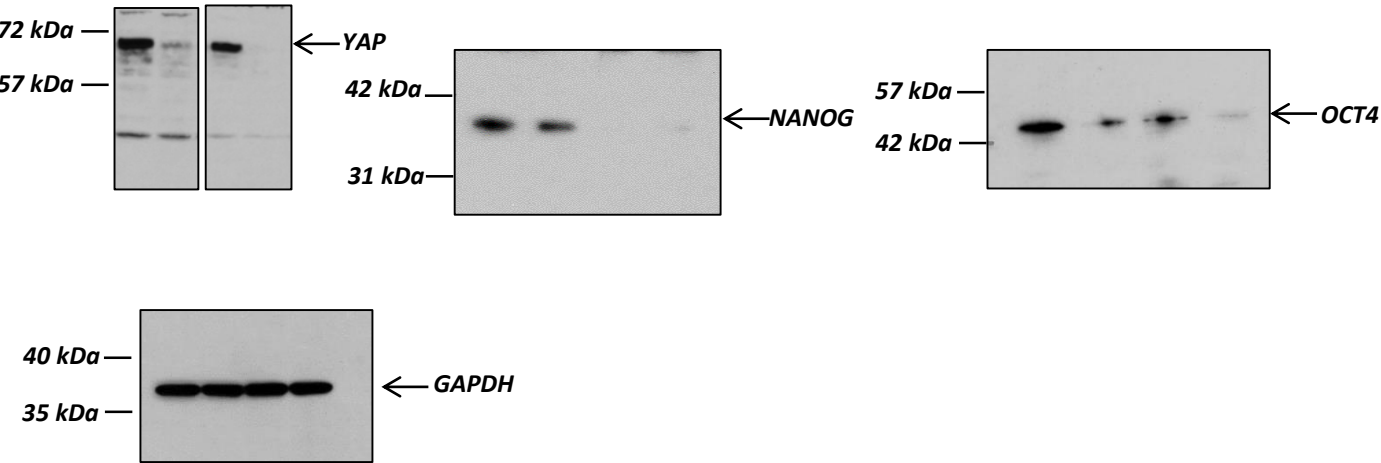

**Figure 6F**

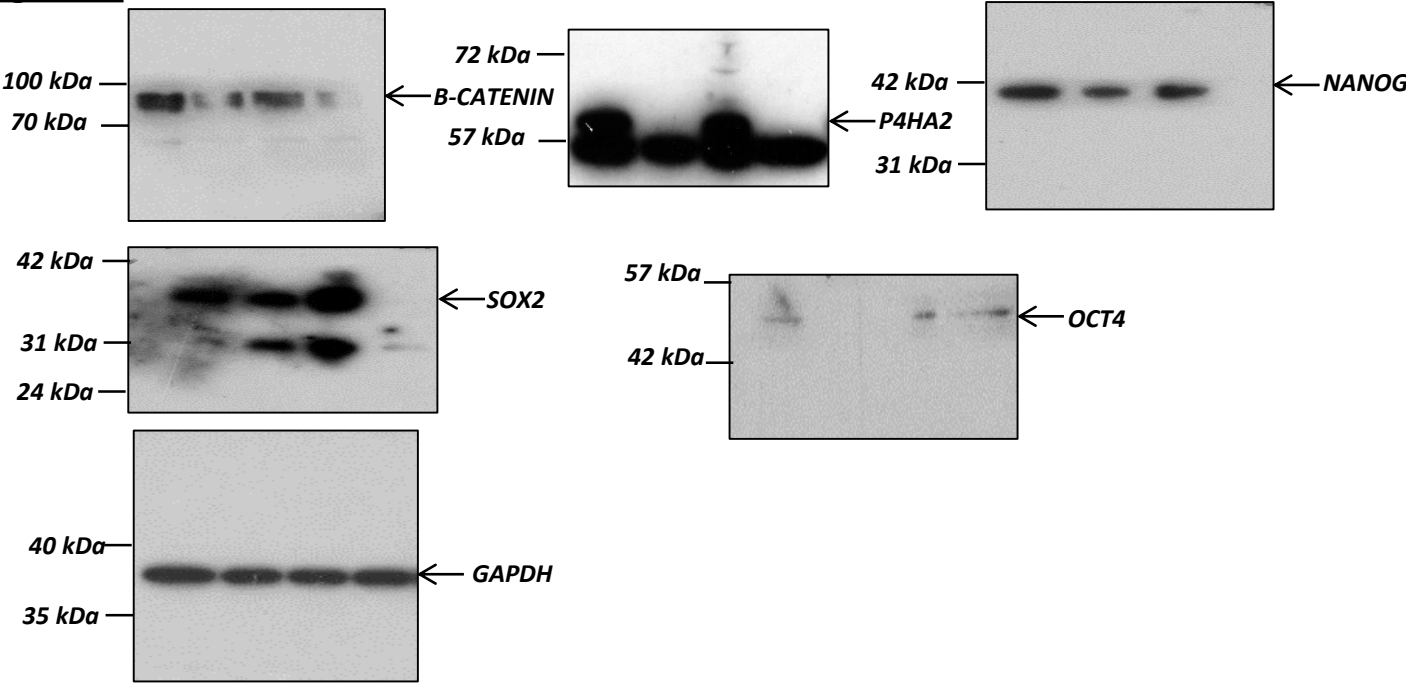

Supplement: Supplementary file 11 — Source Data for Figure 6 [file EMBJ-38-e100532-s009.pdf]

**Figure 7B**

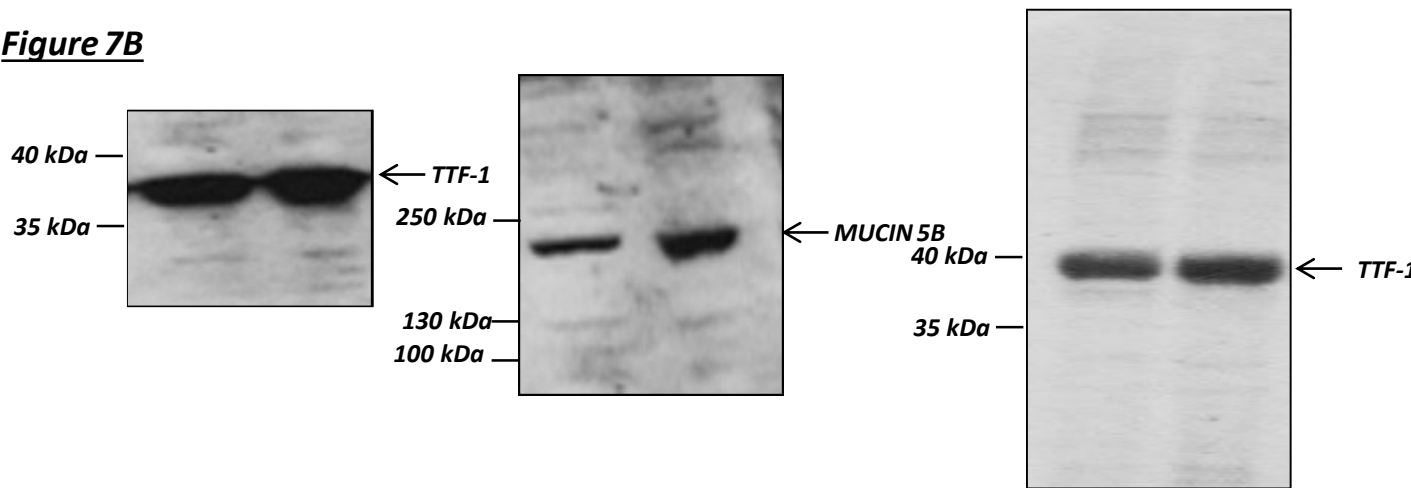

Supplement: Supplementary file 12 — Source Data for Figure 7 [file EMBJ-38-e100532-s010.pdf]
